# The Treatment of Advanced Breast Cancer by Hormone Therapy

**DOI:** 10.1038/bjc.1952.4

**Published:** 1952-03

**Authors:** Mary Douglas

## Abstract

**Images:**


### THE TREATMENT OF ADVANCED BREAST CANCER BY HORMONE THERAPY.

MARY DOUGLAS.

*From the Radiotherapy Department, Royal Infirmary, Edinburgh.*

Received for publication December 18, 1951.

THE physiological relationship between the breast and the ovary has long been recognised, and as knowledge of this subject has advanced, it has been applied with increasing benefit to the treatment of advanced carcinoma of the breast.

Since the first suggestion by Schinzinger at the Surgical Congress in Germany in 1889 (Schinzinger, 1889) and the first work by Beatson published in 1896, bilateral oöphorectomy has been tried as an auxiliary method of treatment of breast cancer. It has been found of value in younger women. With the advent of X-rays the same effect was brought about by ovarian irradiation (Foveau de Courmelles, 1926 ; Ahlbom, 1930).

On the experimental side, the work of Lathrop and Loeb (1916), Loeb (1919) and Murray (1928) demonstrated the part played by the ovary in the production of mammary tumours, while Lacassagne (1932) showed that, in certain circumstances, mammary carcinomata could be induced by the injection of oestrogenic substances.

Interest in the problem was further stimulated by the work of Huggins and Hodges (1941) on the treatment of carcinoma of the prostate by castration and stilboestrol.

It was logical that the next step in the management of advanced breast cancer in women should be the use of androgens. Testosterone propionate was tried with success by Ulrich (1939) and by many later workers.

The treatment of women suffering from breast cancer with one of the oestrogen preparations was apparently illogical, although Zondek (1936) had put forward the suggestion in 1936. He pointed out that such treatment would bring about an inhibition of the gonadotrophic and growth hormones of the anterior pituitary, and that this, in turn, would produce a " medical castration " and possibly growth restraint in the tumour.

The next mention in the literature of work of this nature was a symposium published in June, 1944 (Ellis *et al.*, 1944). The work appears to have been started independently in various centres as a result of the growing interest in the relationship between malignant disease and sex hormones. The results amply proved the value of the oestrogens.

### *Type of case receiving hormone treatment.*

Since 1940 various types of hormone therapy have been used in this Department. Such treatment was given to patients who were beyond the aid of surgery and radiotherapy or who had recurred following orthodox methods of treatment. In these recurrent cases sufficient time had been allowed to elapse, so that previous treatment did not influence the results obtained from hormone therapy.

The present series of 656 cases includes patients treated by ovarian irradiation, by surgical castration accompanied by the administration of testosterone propionate, by various oestrogen preparations (stilboestrol dipropionate, dienoestrol, triphenylchlorethylene and ethinyl oestradiol), by ethisterone and by testosterone propionate alone. It also includes a group treated by irradiation of the pituitary.

*Assessment of response.*

Assessment of response to the various methods of treatment was based entirely on the observed regression of tumour tissue, and improvement in general health alone was disregarded because such drugs as stilboestrol and testosterone are known to produce this effect in patients not suffering from malignant disease.

Spontaneous regression of tumours has occasionally been reported (Ewing 1940). They are rare, however, and in over 4000 cases of breast cancer observed in this Department no spontaneous regression has so far been noted.

The survival rate will be mentioned in the groups of cases to be considered and, although it seems that life can be prolonged with hormone treatment, no attempt is made to claim success on this account alone.

The responses were graded as follows :

A. *Good response.*—There was complete disappearance of soft tissue metastases or radiological evidence of new bone formation in skeletal metastases.

B. *Fair response.*—There was regression of soft tissue metastases without complete disappearance, or relief of pain in skeletal metastases.

C. *No response.*—There was steady progression of the disease.

These three groups, A, B and C, applied to the series treated by ovarian or pituitary irradiation. Further types of response were noted where hormones were administered :

D. *Adverse response.*—The course of the disease appeared to be accelerated.

E. *Withdrawal response.*—No response was noted during administration of the hormone, but a beneficial effect was produced after the drug had been stopped. This effect has been noted by others (Farrow, 1944). It may be due to a delayed response and the point would require further investigation.

*Patients Treated by Ovarian Irradiation.*

Treatment by ovarian irradiation was begun in May, 1940, and the results for all patients treated up to June, 1950, have now been assessed.

The technique of treatment has varied slightly over the years, but the aim was to deliver to the ovaries a dose of about 700 to 800r over a period of one week. Where the patient lived some distance away, ovarian irradiation was carried out in a single treatment.

Women of all ages were treated to determine the effect in the different age-groups. Amongst younger women, the dose was sufficient to bring about a cessation of menstruation in all cases.

From a consideration of Table I it will be seen that about 20 per cent of all patients showed a favourable response.

It will be noted too that the average survival rate after treatment by ovarian irradiation was longer in the groups showing a good response. This has to be interpreted carefully because (*a*) there were many individual exceptions, and (*b*) the natural course of the disease is very variable. The difference between the average survival in groups A and C is so great, however, that even bearing these qualifying facts in mind, there seems to be no doubt that life can be prolonged on occasions by ovarian irradiation.

TABLE I.—*Results following Ovarian Irradiation.*

Total number of cases = 175.

| Response. | Numbers. | Average survival/mths. | Average age/yrs. | Percentage premenopausal. |
|---|---|---|---|---|
| A. Good | 17 (9·7%) | 36·2 | 43·1 | 94·1 |
| B. Fair | 19 (10·9%) | 19·2 | 44·1 | 94·7 |
| C. None | 139 (79·4%) | 10·1 | 49·7 | 48·2 |
| Total | 175 (100·0%) | | | |

As a general rule the best response occurred in women who had not reached the menopause. Indeed, if premenopausal patients are considered as a separate group, it is found that approximately 32 per cent showed a response of some degree.

Each case was carefully tabulated according to the response shown and according to the histological type of tumour, but no correlation between the two could be found. All histological types of tumour showed a good response on occasions.

An attempt was also made to correlate the degree of response with the site of the metastases, but, here again, no correlation was found. Good responses were seen in all types of metastases, skin nodules, glands, pulmonary and skeletal metastases. In 3 patients with osteolytic bone metastases there was radiological evidence of new bone formation. In 4 a pleural effusion disappeared and in 4 it diminished in amount. One patient showed regression of orbital metastases.

A short account is given of 2 of the patients showing a good response:

*Case No.* 26,101, *Mrs. E. T*—.—This patient was first seen on 27.vii.46 when she was 39 years of age and about 4 months pregnant. She had a carcinoma of the left breast and widespread osteolytic skeletal metastases. She was treated by simple mastectomy and palliative X-ray therapy to the region of the acetabula. Two months later (15.ix.46) a film of the pelvis (Fig. 1) showed that there was still very extensive metastatic involvement of the acetabular regions, and on this account a caesarean section was performed on 11.x.46. Fig. 2 shows the appearance of the pelvis on 29.x.46. Following this she had ovarian irradiation but the whole pelvis was not irradiated. New bone formation occurred in parts of the skeleton beyond the treated area. Fig. 3 illustrates the remarkable response that had occurred in the pelvis by 14.iv.47. Her general health improved immensely and she became pain-free, but later her condition deteriorated and she died on 3.vii.47.

*Case No.* 17,361, *Mrs. C. S*—.—This case illustrates the remarkable reduction that may occur in a pleural effusion. She was originally treated by simple mastectomy and X-ray therapy in 1943 for a Stage I carcinoma breast when she was 38. In November, 1945, she developed a right-sided pleural effusion as shown in Fig. 4. She received ovarian irradiation and the effusion gradually diminished in amount. Fig. 5 illustrates the findings on 9.x.46. After this her general condition became poorer and she died in February, 1948.

*Patients Treated by Surgical Castration and Testosterone.*

In 1941 a small group of patients was treated by surgical castration, accompanied by the administration of testosterone. This was done in an attempt to find out why there were some failures with ovarian irradiation. It was thought that ovarian function might recover to some extent after irradiation and that the effect of oöphorectomy would be more lasting.

Nine patients with advanced breast carcinoma were treated in this manner by oöphorectomy, followed by daily intramuscular injections of 25 mg. testosterone propionate for periods up to 3 months.

Only one patient showed a slight response. She was a premenopausal woman of 40 with an inoperable breast carcinoma and bone metastases. The primary tumour became smaller and the glands disappeared temporarily, but no effect was noted on the skeletal metastases.

*Patients Treated by Oestrogen Therapy.*

In the latter part of 1942 it was decided to investigate the value of oestrogens in advanced breast carcinoma, and since that time the following oestrogens have been tried: stilboestrol dipropionate, dienoestrol, triphenylchlorethylene and ethinyl oestradiol. The drugs were given to women of all ages.

*Stilboestrol dipropionate.*

The dose used in the majority of patients was 5 mg. twice a day. This level of dosage produced only slight gastro-intestinal upset as a rule, and appeared to be adequate for the production of a good response. In a few patients where a gastro-intestinal upset was troublesome the dose was reduced to 2·5 mg., 1 mg. or even 0·5 mg. twice a day.

In patients with a pleural effusion, 5 mg. twice a day sometimes caused sufficient water retention to increase the pleural effusion and add to the patient's dyspnoea. It was remarkable to find in a few such patients that doses as small as 0·5 mg. twice or thrice a day were effective in causing a marked reduction or even disappearance of the effusion.

The drug was given for an initial period of 6 to 8 weeks and then on alternate months. Administration was continued indefinitely where a good response was obtained, even although in some cases the patient became clinically free from disease. This intermittent form of dosage seemed to prolong the good effect, and also minimised endometrial hyperplasia and the risk of withdrawal menorrhagia. Slight vaginal bleeding or discharge occurred intermittently in most patients.

The incidence of toxic symptoms was not unduly high. The number of patients having no symptoms attributable to the hormone while taking stilboestrol was 122, while many of the remainder had symptoms which did not inconvenience them greatly, the most common being nausea and vomiting at the commencement of treatment. Many complained of frequency of micturition and incontinence. Only 8 showed complete intolerance of the drug when given at a lower dosage.

It can be seen from a consideration of Table II that there was quite a gratifying response—approximately 30 per cent showing some degree of improvement.

The relation between response and the time of occurrence of the menopause is as striking as in the group treated by ovarian irradiation, only the reverse obtains here—the best results are seen after the menopause. There is also an exceptionally high proportion of adverse responses among premenopausal women. This is not a rigid rule, however, as there were adverse responses in older women (the oldest was over 70) and there were fair responses in young women.

TABLE II.—*Results following Treatment with Stilboestrol Dipropionate.*

Total number of cases = 322.

| Response. | Numbers. | Average survival/mths. | Average age/yrs. | Percentage premenopausal. |
|---|---|---|---|---|
| A. Good | 33 (10·3%) | 30·5 | 66·3 | 0 |
| B. Fair | 65 (20·2%) | 19·9 | 62·9 | 9·2 |
| C. None | 183 (56·8%) | 9·1 | 57·9 | 17·5 |
| D. Adverse | 33 (10·3%) | 6·3 | 54·7 | 33·3 |
| E. Withdrawal | 8 (2·4%) | 27·3 | 58·1 | 12·5 |
| Total | 322 (100·0%) | | | |

Again, as a general rule, those who showed a good response survived longer, although there were several notable exceptions.

As in the group treated by ovarian irradiation, no correlation was found here between response and the histological type of the tumour.

In general, where a good response occurred, all metastases showed improvement. However, in some patients one metastasis might regress while another became larger, or new ones appeared.

All types of metastases were found to respond on occasions—skin nodules, glands, lung and orbital metastases. Even pleural effusions dimished in amount, and in 3 cases disappeared altogether with no treatment other than stilboestrol dipropionate. It is also very interesting to note that in this series 13 patients with bone metastases experienced relief from pain, while in 6, new bone formation was demonstrated radiologically in osteolytic lesions. As this is not a finding generally reported it may be useful to illustrate it with the following case:

*Case No.* 11,124, *Mrs. E. B*—.—This patient had a radical excision of the right breast for carcinoma in 1928 when she was 43. She kept well until October, 1944, when she developed proptosis of the left eye. She was seen in the Radiotherapy Department for the first time on 24.v.45, when she was found to have metastases in the left orbit, enlarged glands in the left side of the neck and diffuse skeletal metastases. Fig. 6 shows the presence of osteolytic and osteoplastic metastases in the pelvis on 24.v.45.

Stilboestrol dipropionate, 5 mg. twice a day, was prescribed, and by 2.viii.45 the proptosis had almost gone and the glands were no longer palpable. The left eye appeared normal apart from slight ptosis of the upper lid. Fig. 7 shows the radiographic appearance of the pelvis on 19.viii.47. There had been considerable improvement, and some of the metastases were difficult to detect.

She continued to take stilboestrol dipropionate 5 mg. twice a day on alternate months and remained well until December, 1948. She developed a tumour of the cardiac end of the stomach and died on 27.viii.49. No post-mortem was carried out, but this gastric tumour was probably metastatic in nature from the carcinoma breast.

The following case illustrates the improvement which may take place in a patient with a pleural effusion:

*Case No.* 127/48, *Mrs. J. L*—.—This patient was treated by simple mastectomy and X-ray therapy in January, 1948, for a Stage I carcinoma of the right breast when she was aged 64. She remained well until November, 1948, when she developed a metastasis in the opposite left breast, which was treated by simple mastectomy and a palliative course of X-ray therapy.

On 19.i.50 she was found to have a right-sided pleural effusion (Fig. 8) extending to the level of the third rib anteriorly. Treatment with stilboestrol dipropionate 0·5 mg. thrice daily was commenced, and by 24.ii.50 there was already considerable reduction in the pleural effusion (Fig. 9). Further reduction took place, until by June, 1950, the findings were within normal limits (Fig. 10). She remained well until October, 1951, when she developed a febrile illness, and an X-ray of her chest showed diffuse pulmonary metastases. Her general condition is now deteriorating rapidly (December, 1951).

*Dienoestrol.*

The dosage of dienoestrol used was 1 mg. thrice daily in most cases. A few received 5 mg. twice a day with no apparent advantage. The incidence of toxic symptoms was minimal. Where a good response occurred the drug was continued indefinitely on alternate months.

The responses are shown in Table III.

TABLE III.—*Results following Treatment with Dienoestrol.*

Total number of cases = 32.

| Response. | Numbers. | Average survival/mths. | Average age/yrs. | Percentage premenopausal. |
|---|---|---|---|---|
| A. Good | 2 (6·3%) | 44·0 | 67·0 | 0 |
| B. Fair | 3 (9·4%) | 9·0 | 60·5 | 0 |
| C. None | 23 (71·8%) | 17·6 | 59·0 | 0 |
| D. Adverse | 4 (12·5%) | 9·5 | 45·8 | 75 |
| E. Withdrawal | 0 | — | — | — |
| Total | 32 (100·0%) | | | |

The results shown in Table III are similar to those obtained in the group treated with stilboestrol in respect of age distribution and relation to the menopause. The numbers treated with dienoestrol are smaller, but a comparison of Tables II and III would tend to suggest that dienoestrol is less effective than stilboestrol.

Of those who did not respond well, one showed a very good and 4 a fair response to stilboestrol.

One patient aged 64 who did not respond to dienoestrol showed a fair response to testosterone. This would seem to suggest that a patient may respond to one type of hormone therapy and not to another, and it would be of interest to pursue this point further.

*Triphenylchlorethylene.*

Only 6 cases were treated with triphenylchlorethylene. The dosage given was 0·05 mg. twice a day. No case was made worse, but only one patient, aged 79, showed a slight response. Glands were reduced in size and lung metastases did not advance for 4 months.

*Ethinyl oestradiol.*

The dosage of ethinyl oestradiol used in the majority of patients was 0·05 mg. twice a day. Out of 38 patients treated, 12 experienced a considerable degree of nausea and vomiting, especially in the first week of treatment. Some complained also of palpitation and vertigo. In 2, the dose had to be reduced to 0·05 mg. a day and, in other 2, it had to be reduced to 0·01 mg. twice a day. This drug has only been used since December, 1948, and it is too soon to assess survival rates.

TABLE IV.—*Results following Treatment with Ethinyl Oestradiol.*

Total number of cases = 38.

| Response. | Numbers. | Average age/yrs. | Numbers who were premenopausal. |
|---|---|---|---|
| A. Good | 2 (5·3%) | 65·0 | 0 |
| B. Fair | 10 (26·3%) | 61·0 | 1 |
| C. None | 24 (63·2%) | 61·5 | 1 |
| D. Adverse | 1 (2·6%) | 44·0 | 0 |
| E. Withdrawal | 1 (2·6%) | 53·0 | 1 |
| Total | 38 (100·0%) | | |

Here again the results obtained are comparable with those shown in Tables II and III.

The general impression gained from a study of Tables II, III and IV is that stilboestrol dipropionate is the oestrogen preparation most likely to give beneficial results. Dienoestrol, however, might be tried in a patient who is markedly upset by stilboestrol.

*Patients Treated by Testosterone.*

A further series of 30 cases was treated with testosterone between July and December, 1949.

The dose used was 50 mg. of testosterone propionate daily, given intramuscularly. Treatment was continued for 3 to 4 weeks, and if the patient's condition showed some improvement, further treatment was given either by means of an implant of 400 to 600 mg. of testosterone propionate into the abdominal wall or by means of methyl testosterone. Methyl testosterone was given at a dose-rate of 50 mg. a day on alternate months, and was administered in the form of tablets which were allowed to dissolve sublingually.

The results are as shown in Table V.

TABLE V.—*Results following Treatment with Testosterone Propionate.*

Total number of cases = 30.

| Response. | Numbers. | Average age (years). |
|---|---|---|
| A. Good | 1 (3·3%) | 50 |
| B. Fair | 7 (23·4%) | 48·2 |
| C. None | 20 (66·7%) | 45·3 |
| D. Adverse | 1 (3·3%) | 43 |
| E. Withdrawal | 1 (3·3%) | 52·1 |
| Total | 30 (100·0%) | |

A remarkable improvement occurred in the general health of about 50 per cent of the patients treated with testosterone, and this feeling of well-being may not be undesirable, especially in the advanced stages of breast carcinoma. It must be emphasised, however, that improvement in general condition was not taken as a criterion of response, and only patients in whom the tumour regressed have been included among those showing favourable responses.

In this group of cases there seems to be no correlation between the type of the response and the age of the patient. Favourable responses occurred in pre- and post-menopausal women, and also in some premenopausal patients who had failed to respond to ovarian irradiation.

The best results in this group occurred in patients with soft tissue metastases. In one, enlarged glands disappeared completely, and in another 2 the primary tumour showed a temporary regression. One patient who was comatose with cerebral metastases improved sufficiently under treatment with testosterone to attempt crossword puzzles. Subsequent post-mortem examination confirmed the presence of large intracerebral metastatic deposits, which presumably had regressed only temporarily under the influence of testosterone.

In another patient a large pleural effusion did not cause any further respiratory embarrassment, and required aspiration only at increasingly longer intervals. This patient is still alive 25 months after the commencement of treatment.

Only one patient experienced relief of pain from skeletal metastases while receiving testosterone. Another patient with metastases in bone stated she felt the pain easier when the drug was withdrawn. There was no radiological evidence of repair occurring in bone metastases. The series, although small, is in this respect at variance with the results so frequently reported in the literature (Adair *et al.*, 1949 ; Kaae, 1949 ; Report of the Council on Pharmacy and Chemistry, 1949).

Only one patient could not tolerate the drug. She was a woman of 43 with cerebral metastases, who developed very severe vomiting, and treatment had to be discontinued after she had received 150 mg. Apart from this one patient no one was upset by treatment. With the dosage used a few complained of nausea at the beginning, but this passed off. Signs of masculinisation were seen in most patients who received 2000 mg. or over. One patient developed a mild acne, which disappeared when the testosterone was stopped. Premenopausal patients developed amenorrhoea.

### *Patients Treated by Ethisterone.*

In order to make the investigation more complete it was felt that the effect of ethisterone (androhydroxyprogesterone) should be tried.

Accordingly it was given to women (*a*) who had not yet reached the menopause, as it was thought that they might show an adverse response to stilboestrol, and (*b*) who had already shown an adverse response to stilboestrol. Actually, only 7 were treated with this drug, but none showed any response.

### *Patients Treated by Pituitary Irradiation.*

In October, 1949, an investigation was begun into the effects of irradiation of the pituitary on the course of breast cancer. It was thought that the treatment would bring about a complete cessation of all oestrogenic activity by suppression of the gonadotrophic hormones. It was hoped that this would produce better results than ovarian irradiation in younger women. Treatment was also given to post-menopausal women to determine the effect.

Many of these patients had already failed to respond to other methods of hormone therapy, and they were beyond the aid of all other forms of treatment.

The pituitary was irradiated by two opposed fields, each 6 × 8 cm. placed in the temporal region, and a dose of 3000r was delivered in 3 weeks to the region of the sella turcica.

A series of 37 patients was treated between October, 1949, and October, 1950, and the results are shown in Table VI.

As will be seen from Table VI, only 2 patients showed a good response. The first patient was a woman of 51 whose periods had stopped 5 months before and who had had no previous hormone treatment. A recurrent mass over the sternum, 6 cm. in diameter, disappeared during the 3 weeks' treatment to the

---

EXPLANATION OF PLATES.

Fig. 1.—Case No. 26,101, Mrs. E. T— (15.ix.46).—Radiograph of pelvis before ovarian irradiation showing extensive osteolytic metastatic involvement, particularly in region of acetabula.

Fig. 2.—Same case as Fig. 1 (29.x.46).—Radiograph of pelvis following caesarean section but before ovarian irradiation was carried out.

Fig. 3.—Same case as Fig. 1 (14.iv.47).—Radiograph of pelvis 5 months after ovarian irradiation showing new bone formation in acetabular regions and rings of sclerosis round osteolytic lesions in ilii.

Fig. 4.—Case No. 17,361, Mrs. C. S— (13.xi.45).—Radiograph of chest showing pleural effusion on right side (before ovarian irradiation).

Fig. 5.—Same case as Fig. 4 (9.x.46).—Radiograph of chest 11 months after ovarian irradiation showing considerable diminution in the amount of the pleural effusion with the presence of a small fibrin body in the right costo-phrenic angle.

Fig. 6.—Case No. 11,124, Mrs. E. B— (24.v.45).—Radiograph of pelvis showing widespread osteolytic metastatic involvement (before administration of stilboestrol).

Fig. 7.—Same case as Fig. 6 (19.viii.47).—Radiograph of pelvis 25 months after commencement of treatment with stilboestrol. There has been considerable new bone formation and restoration of the normal trabecular pattern.

Fig. 8.—Case No. 127/48, Mrs. J. L— (19.i.50).—Radiograph of chest showing large pleural effusion on right side (before treatment with stilboestrol).

Fig. 9.—Same case as Fig. 8 (24.ii.50).—Radiograph of chest 1 month after commencement of treatment with stilboestrol. There has been some diminution in amount of the pleural effusion.

Fig. 10.—Same case as Fig. 8 (16.vi.50).—Radiograph of chest showing complete disappearance of pleural effusion.

1

2

Douglas.

3

4

5

6

7

Douglas.

Douglas.

TABLE VI.—*Results following Pituitary Irradiation.*

Total number of cases = 37.

| Response. | Numbers. | Average age (yrs). |
|---|---|---|
| A. Good | 2 (5·4%) | 48·5 |
| B. Fair | 2 (5·4%) | 54·0 |
| C. None | 33 (89·2%) | 52·9 |
| Total | 37 (100·0%) | |

pituitary in October, 1950. She remained well until August, 1951, when she developed skeletal metastases, and her general condition is now very poor (December, 1951).

The second good response occurred in a woman of 46. She had had an artificial menopause brought about by ovarian irradiation one year previously in an attempt to control a local recurrence of the tumour. The recurrence had become flatter for 3 months, but after that had spread again. Following pituitary irradiation complete healing took place slowly, but new nodules appeared 10 months later.

Of the 2 women showing a fair response, the first was aged 46 and premenopausal. She had a skin recurrence on the chest wall within the irradiated area and was treated by pituitary irradiation in December, 1949. Slight regression of the recurrence was noted for 3 months, but then the disease became widespread and she died on 6.xii.50. She had no periods following pituitary irradiation. The other patient showing a fair response was aged 62 and had had a normal menopause 7 years previously. In December, 1949, she developed oedema and congestion of the face and neck due to pressure from enlarged mediastinal glands. She was treated with pituitary irradiation and her symptoms were relieved during treatment. Unfortunately, one month later she developed other metastases and her condition deteriorated. She died on 6.vi.50.

Among the 33 who did not respond the ages varied from 30 to 73. Twenty-two had had ovarian irradiation previously, 6 had had stilboestrol and 2 testosterone. None had shown any response to the other types of hormone treatment. It would seem from this that pituitary irradiation may be of value in the type of case suitable for ovarian irradiation, or in cases where ovarian irradiation has already been given with some good temporary effect.

There is no doubt that pituitary irradiation was the least well tolerated of all forms of hormone therapy. Fourteen patients were very upset by the treatment before the course was completed. It is possible that a modification of technique and dosage would overcome some of these difficulties.

Of the 37 patients, 21 have died within one year, the average survival being 2 to 9 months after the commencement of treatment. Of the remaining 16, only 5 are now able to be up and about.

*Comparison with Cases Reported in the Literature.*

The results which have been obtained agree with those already reported in the literature as far as response in relation to age and the date of occurrence of the menopause are concerned. There are, however, several interesting points of difference, and some facts which have not been specially noted before.

In the first place, although many series of cases have been published in the past emphasising the value of ovarian irradiation in the treatment of premenopausal women with advanced breast cancer (Foveau de Courmelles, 1926; Ahlbom, 1930; Dresser, 1936; Martin, 1936; Smith, 1936), this particular method appears to have been neglected in recent years in favour of testosterone. The findings in Edinburgh suggest that it might with advantage be more extensively used. It is very simply given as a single treatment in an X-ray department, whereas treatment with testosterone is prolonged and expensive. It is true that some women have troublesome side-effects such as flushings after ovarian irradiation, but the results of prolonged testosterone administration, hirsutes and other signs of masculinisation are not less distressing.

All writers are agreed on the value of the oestrogens in post-menopausal women (Ellis *et al.*, 1944; Haddow, Watkinson and Paterson, 1944; Taylor, *et al.*, 1948; "Report of the Council on Pharmacy and Chemistry," 1949; Adair, *et al.*, 1949; Walpole and Paterson, 1949). However, most reports state that the best responses occur in patients with soft tissue metastases. It is generally considered that oestrogens are of little value in the treatment of skeletal metastases, although Paterson (1944) reported relief from pain in such cases. In the present series it was found that response was as likely to occur in bone as elsewhere and, indeed, in 6 cases there was radiological proof of repair occurring in osteolytic metastases.

Conversely, our experience here with testosterone has been that almost all the favourable responses have occurred in extra-skeletal metastases. No good results were seen in bone lesions apart from relief of pain in 2 cases, although, of course, the series was small, and treatment may not have been continued over a long enough period. The reports in the literature on the efficacy of testosterone are very variable. All gradations of response have been reported. Farrow (1944) noted a deleterious effect in female patients, Nathanson (1944) observed no effect at all, and various later workers (Adair, 1947; Adair *et al.*, 1949; Kaae, 1949) reported a proportion of good responses in skeletal and extra-skeletal metastases. All are agreed that testosterone may produce a beneficial effect on the general health but this is a non-specific effect, and due partly to the stimulating effect of the hormone on protein metabolism. It is, however, important not to assess improvement in general health as an anti-carcinogenic effect.

With both oestrogen and androgen therapy some responses were noted here after the drug was stopped—a withdrawal response. Farrow (1944) also noted this occasionally in the metastases after withdrawal of either oestrogens or androgens.

### *Consideration of the Mode of Action of Hormone Therapy.*

While hormone therapy is widely employed, the basis of treatment remains largely empirical. The extension of our knowledge of the exact mode of action would be of great interest, and might result in further advances in this form of therapy. The clinical results obtained, however, appear to be due to some modification of the endocrine status of the patient.

For example, in a young patient who has not reached the menopause, oöphorectomy or ovarian irradiation causes a sudden withdrawal of the natural ovarian hormones which influence the rate of growth of the cells of breast tissue whether these cells are normal or malignant. The withdrawal of these naturally occurring oestrogenic substances results in an increased output of the gonadotrophic hormones of the anterior pituitary which stimulate extra-gonadal sources of oestrogenic activity such as the suprarenal cortex. Thus, as time passes, the hormone balance of the body is partly restored and the beneficial effects of castration wear off.

Pituitary irradiation must produce a similar effect to ovarian irradiation in younger women, and bring about a cessation of all natural oestrogenic function. The temporary effect obtained in the cases treated might, therefore, be due to the level of dosage employed. At this stage of our knowledge, however, it is doubtful if it would be justifiable to use a higher dosage.

The administration of an artificial oestrogen again modifies the endocrine status. The oestrogens may produce a direct effect on the breast cancer cells, or the effect may be obtained indirectly through the anterior pituitary.

If the action is a direct one, the best results would be expected in older women. In an older patient, for example a woman who is 5 or more years past the menopause, there is little or no circulating oestradiol, and the addition of oestrogens produces a change. After the passage of time, however, the body, and in particular the breast cells, may become readjusted, and this might explain why the good effect passes off.

The work of Zondek suggests that the oestrogens may have an indirect effect through the anterior pituitary. Zondek (1936) found that the administration of large doses of oestrin produced a chromophobe adenoma of the pituitary in male rats, that is, in rats not normally subject to the action of large amounts of oestrogen.

Zondek (1947) also reports a case in which he gave large doses of oestradiol benzoate to a woman of 26 with metastases from a carcinoma breast. No effect was noted on the metastases, but after death her pituitary was found to weigh 710 g. as compared with a normal of 595 g. (the average for a nullipara of her age according to Zondek). The increase in size of the pituitary was due to an eosinophil adenoma.

Unless the production of an adenoma of the pituitary interferes with the output of other hormones the explanation is not very satisfactory in older patients.

In a premenopausal patient, a drug such as stilboestrol may cause inhibition of the gonadotrophic hormones, so suppressing natural ovarian secretion, but not bringing about a true castration. Castration could not be said to be complete while an artificial oestrogenic substance is circulating in the blood. Consequently, there is not the same profound change in the endocrine status and no useful therapeutic effect is produced.

The question of the administration of testosterone might now be considered. In a premenopausal patient this brings about an artificial menopause and, from this point of view, testosterone might be expected to produce similar results to ovarian irradiation or oöphorectomy. In addition to this, the administration of testosterone must disturb the normal oestrogen-androgen ratio. Finally, testosterone, like the oestrogens, causes inhibition of the gonadotrophic hormones of the anterior pituitary (Moore and Price, 1932). It may, therefore, like the oestrogens, produce an indirect effect on the breast cells by a disturbance of pituitary function.

An explanation must now be sought for the failures. By any method so far outlined the successes do not constitute more than about 30 per cent of the total. It is possible that some patients have a more stable endocrine system, due to more rapid metabolic processes which quickly counteract the effects of therapeutically induced changes. In this connection the liver must play an important part, because the liver inactivates ovarian oestrogens (Golden and Severinghaus, 1938). In hepatic cirrhosis there is an excess of free oestrogenic substances in the blood. Westerfeld (1940) says tyrosinase, on incubation, inactivates oestrone, oestradiol and diethylstilboestrol. Zondek and Sklow (1942) believe the inactivation is caused by an enzyme—oestrinase—similar to but not identical with tyrosinase. Jailer (1948) found that in mice fed on a diet deficient in vitamin $B_1$, the degradation mechanism of oestrogens in the liver was impaired. It was not the vitamin $B_1$ deficiency but the concomitant inanition which was found to be the cause. In addition to the more common causes of liver dysfunction, patients with carcinoma of the breast may have metastatic involvement of the liver. How this would affect the response is not known exactly, but it is perhaps significant that few patients with clinical evidence of liver involvement showed any response to any of the methods of treatment mentioned.

There may, however, be other factors modifying the response. The type of tumour may play a part. Haddow, Watkinson and Paterson (1944) suggested that the tumour called " carcinoma of the breast " might in reality comprise several categories. Walpole and Paterson (1949) also made the same suggestion, even going so far as to say that a tumour might produce an " anti-hormone " or inhibitory factor antagonising the action of the oestrogen. They supported this theory by their findings that, in general, patients who did not respond to oestrogen therapy did not show such a complete degree of keratinisation of the vaginal epithelium. If this change in the original epithelium is not obtained in a post-menopausal woman, they suggested that some inhibitory factor was at work. It seemed as though some substances were being produced, either in the tumour or elsewhere, which were able to nullify oestrogenic activity.

A great deal of further work will be necessary before the exact mode of action can be determined.

### SUMMARY AND CONCLUSIONS.

An extensive series of cases of advanced breast carcinoma treated by hormone therapy has been surveyed and the results obtained have been presented.

The findings suggest that, in premenopausal women, the treatment of choice is ovarian irradiation, which was found to produce beneficial results in 30 per cent of patients in this category.

In women who are 5 or more years past the menopause, oestrogen therapy would appear to be the most satisfactory form of treatment. The drug found to be most generally useful was stilboestrol dipropionate 5 mg. twice a day on alternate months.

While the results of treatment with testosterone have been disappointing, it would appear to be the most appropriate method of treatment for post-menopausal women who are not more than 5 years past the menopause, and it should be noted that responses can be obtained where the metastases are confined to soft tissues.

Pituitary irradiation is no longer used routinely, because of the marked systemic disturbance produced. It may be of value in cases treated by ovarian irradiation where the good effect has passed off.

It should be noted that both oestrogens and androgens promote water retention, and in a patient with a pleural effusion the dose has to be greatly reduced,

Good results were noted with doses of stilboestrol dipropionate as low as 0·5 mg. twice a day.

While ordinary methods of histological examination have failed to show any correlation between histological type of the tumour and the response to hormone therapy, it is possible that there are some differences in the cell which are not detected by present methods.

Several suggestions have been offered to explain the mode of action, but much work requires to be done on this aspect of the subject before further advances on the therapeutic side are possible.

In conclusion, I would like to express my thanks to Professor Robert McWhirter for his help and advice in the treatment of these cases and in the preparation of this paper.

### BIBLIOGRAPHY.

Adair, F. E.—(1947) *Surg. Gynaec. Obstet.*, **84**, 719.
*Idem*, Mellors, R. C., Farrow, J. H., Woodard, H. Q., Escher, G. C., Urban, J. A. *et al.*—(1949) *J. Amer. med. Ass.*, **140**, 1193.
Ahlbom, H.—(1930) *Acta Radiol.*, **11**, 614.
Beatson, G. T.—(1896) *Lancet*, ii, 104.
Dresser, R.—(1936) *Amer. J. Roentgenol*, **35**, 384.
Ellis, F., Adams, S. B., Blomfield, G. W., Haddow, A., Levitt, W. M., McWhirter, R., Paterson, E., Thurgar, C. J. L., Walker, J. Z., Windeyer, B. W. *et al.* —(1944) *Proc. Roy. Soc. Med.*, **37**, 731.
Ewing, J.—(1940) 'Neoplastic Diseases.' London (W. B. Saunders), 4th ed., p. 587.
Farrow, J. H.—(1944) *Surgery*, **16**, 141.
Foveau de Courmelles—(1926) *Acta Radiol.*, **6**, 322.
Golden, J. B., and Severinghaus, E. L.—(1938) *Proc. Soc. exp. Biol., N.Y.*, **39**, 361.
Haddow, A., Watkinson, J., and Paterson, E.—(1944) *Brit. med. J.*, ii, 393.
Huggins, C., and Hodges, C. V.—(1941) *Cancer Res.*, **1**, 293.
Jailer, J. W.—(1948) *Endocrinology*, **43**, 78.
Kaae, S.—(1949) *Acta Radiol.*, **31**, 97.
Lacassagne, A.—(1932) *C. R. Acad. Sci.*, Paris, **195**, 630.
Lathrop, A. E. C., and Loeb, L.—(1916) *J. Cancer Res.*, **1**, 1.
Loeb, L.—(1919) *J. med. Res.*, **40**, 477.
Martin, C. L.—(1936) *Amer. J. Roentgenol.*, **36**, 314.
Moore, C. R., and Price, D.—(1932) *Amer. J. Anat.*, **50**, 13.
Murray, W. S.—(1928) *J. Cancer Res.*, **12**, 18.
Nathanson, I. T.—(1944) *Surgery*, **16**, 108.
Paterson, E.—(1944) *Proc. Roy. Soc. Med.*, **37**, 733.
"Report of the Council on Pharmacy and Chemistry"—(1949) *J. Amer. med. Ass.*, **140**, 1214.
Schinzinger.—(1889) *Verh. dtsch. Ges. Chir.*, **18**, 28, quoted by Halberstaeder, L., and Hochman, A.—(1946) *J. Amer. med. Ass.*, **131**, 810.
Smith, E. G.—(1936) *Amer. J. Roentgenol.*, **36**, 65.
Taylor, S. G., Slaughter, D. P., Smejkal, W., Fowler, E., and Preston, F. W.—(1948) *Cancer*, **1**, 604.
Ulrich, P.—(1939) *Acta Unio int. Cancr.*, **4**, 377.
Walpole, A. L., and Paterson, E.—(1949) *Lancet*, ii, 783.
Westerfield, W. W.—(1940) *Biochem. J.*, **34**, 51.
Zondek, B.—(1936) *Lancet* i, 776.—(1947) *Acta Radiol.*, **28**, 433.
*Idem* and Sklow, J.—(1942) *Proc. Soc. exp. Biol., N.Y.*, **49**, 629.


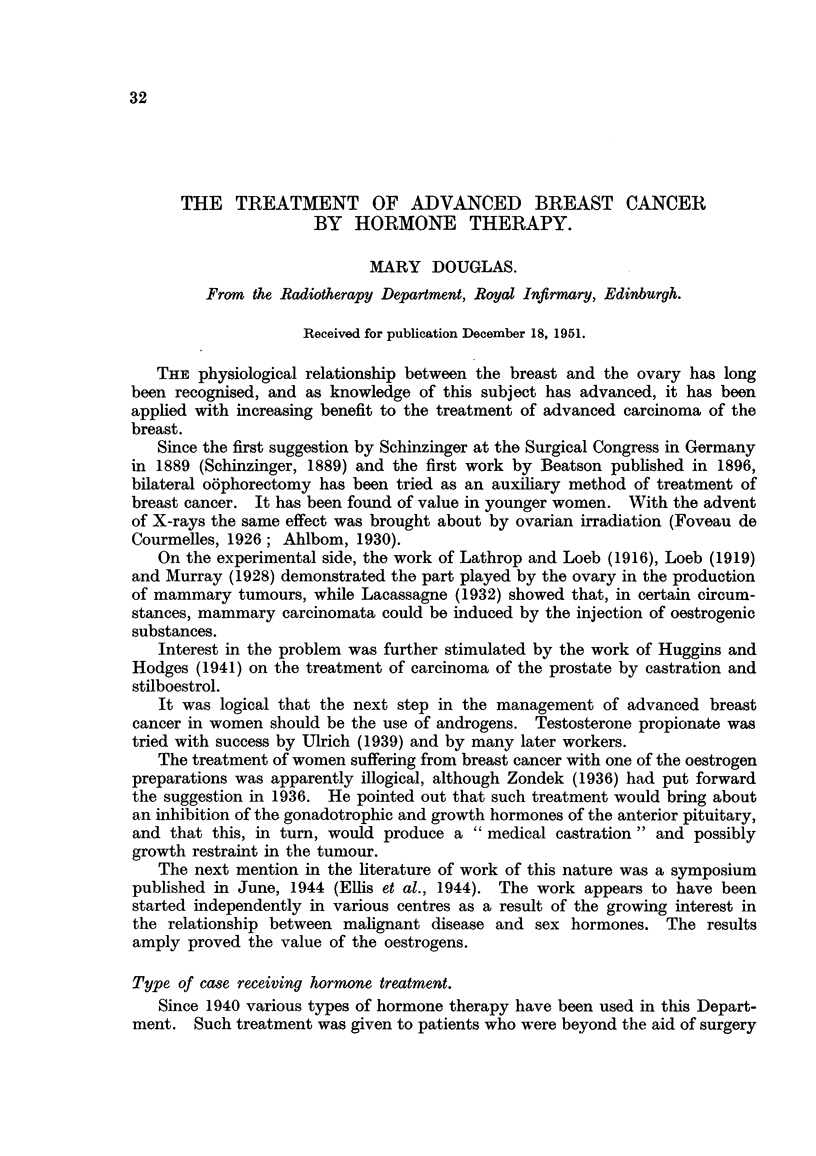

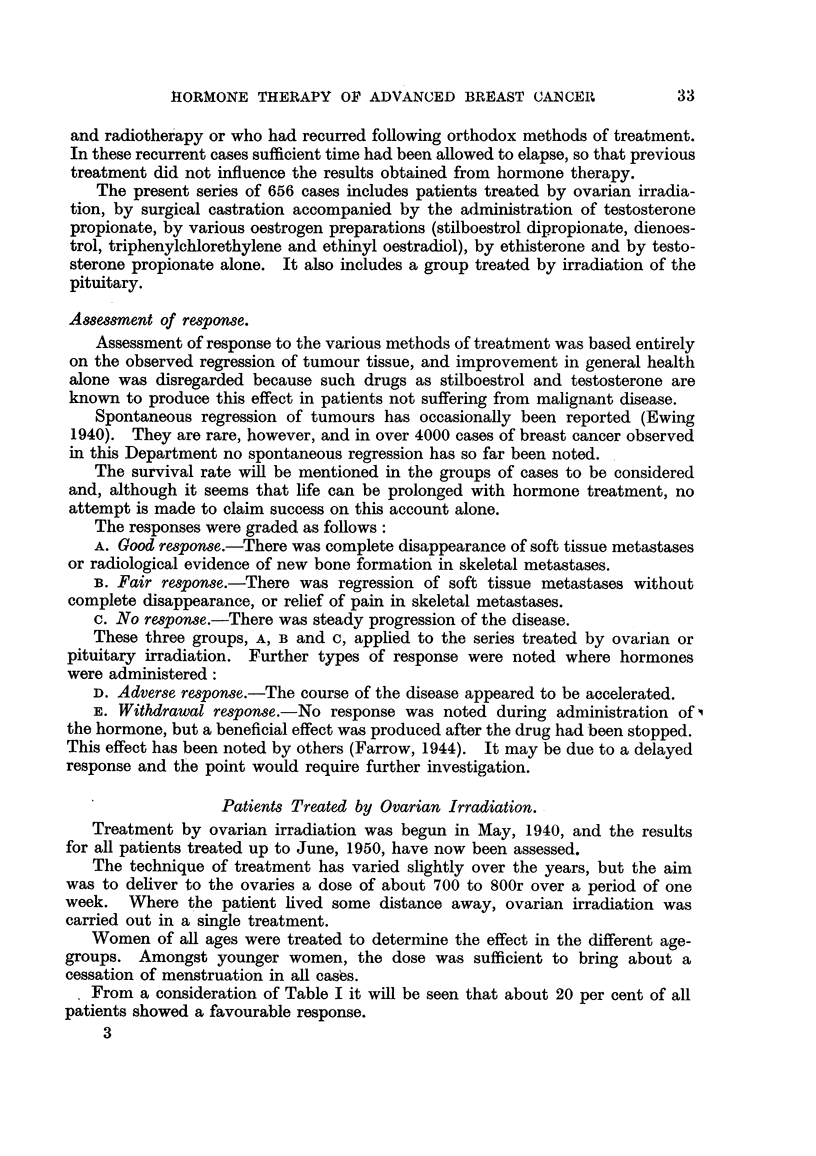

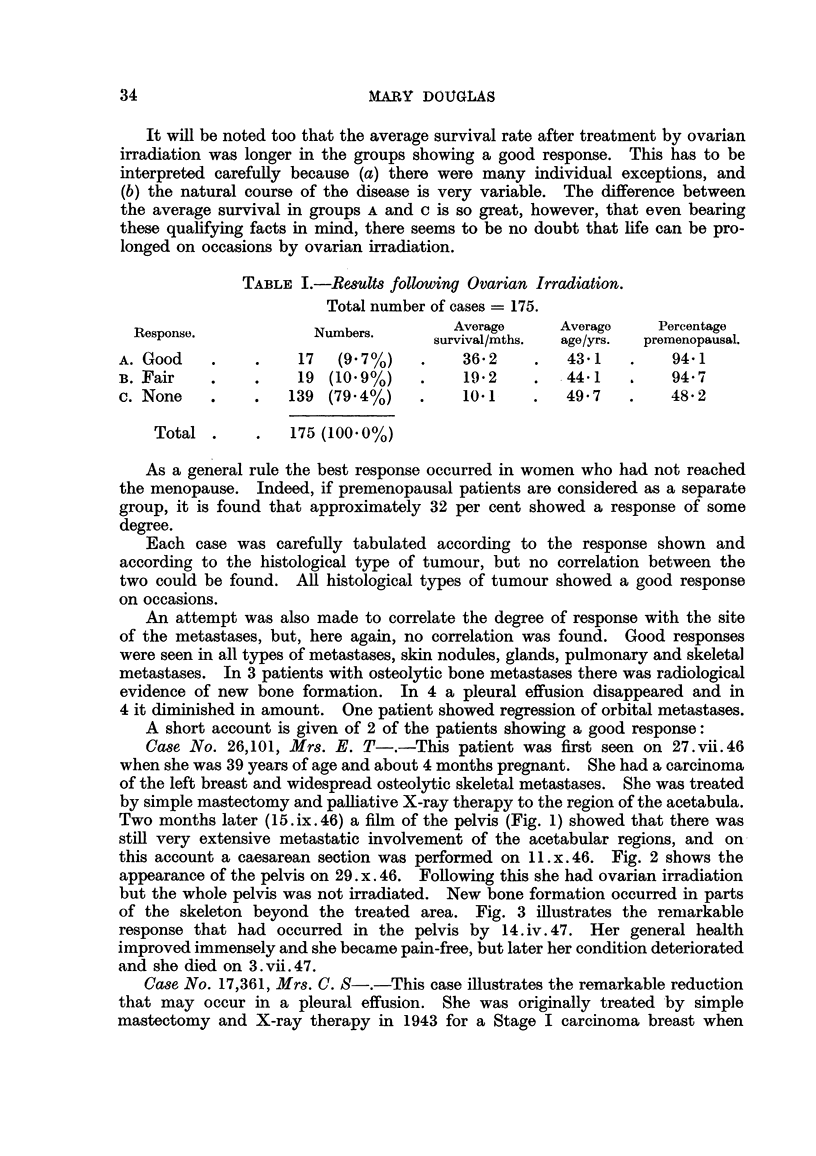

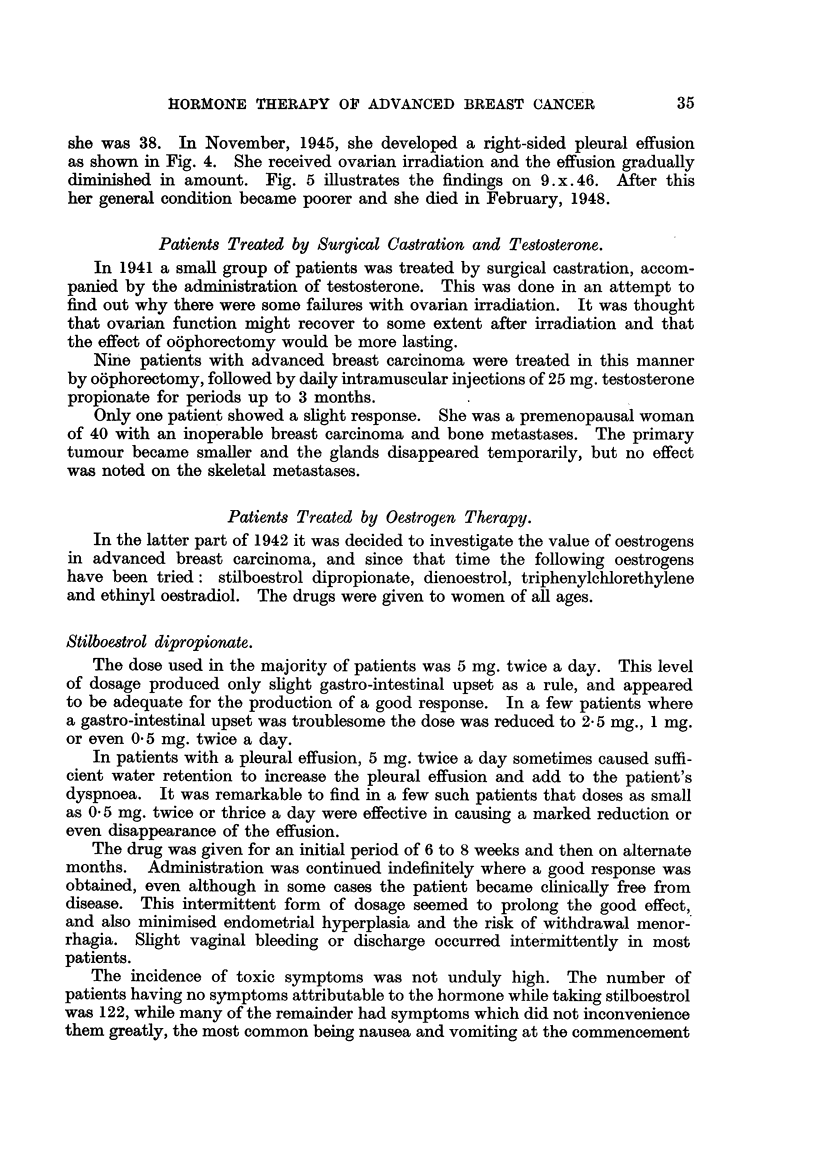

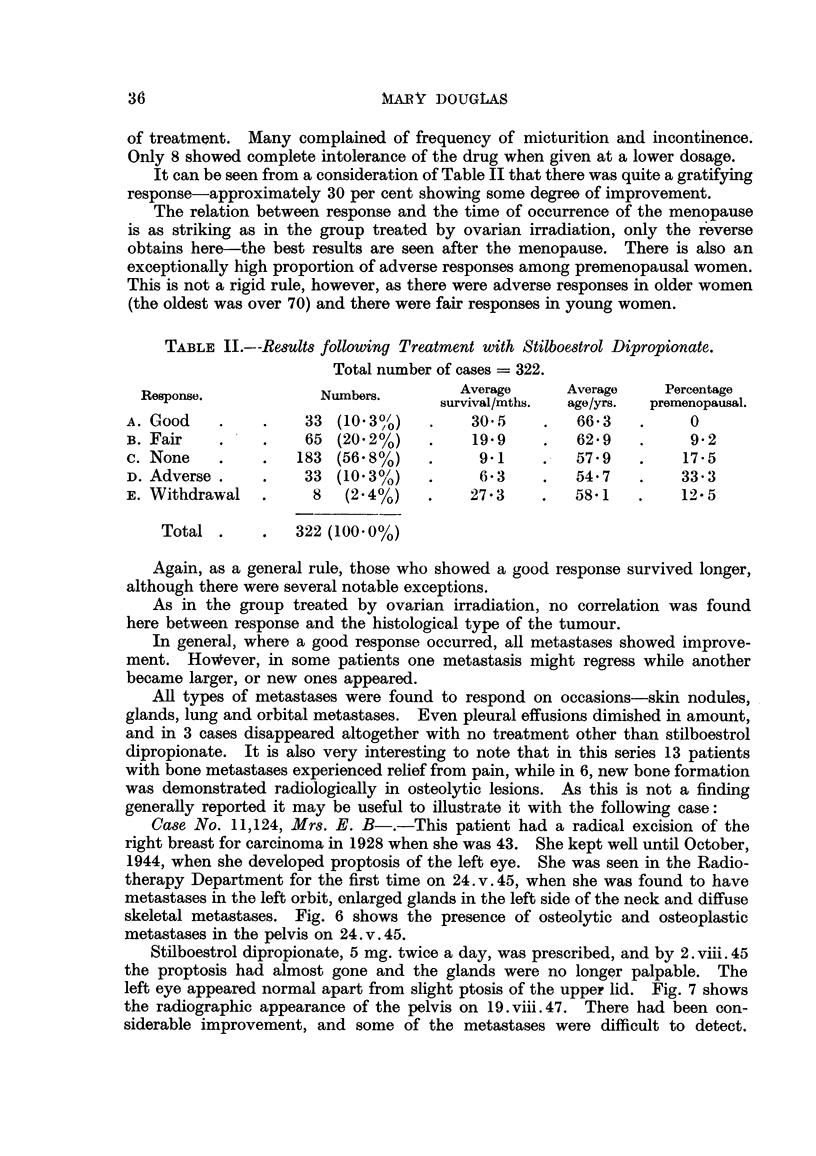

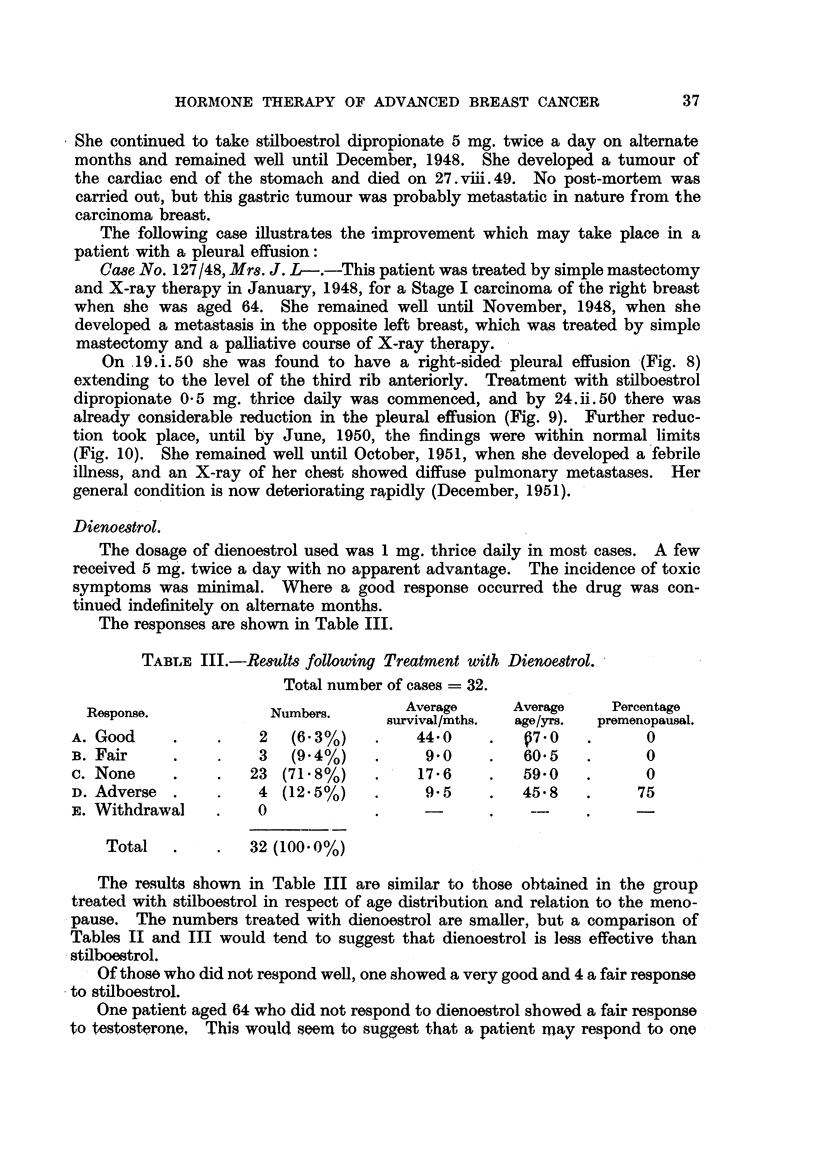

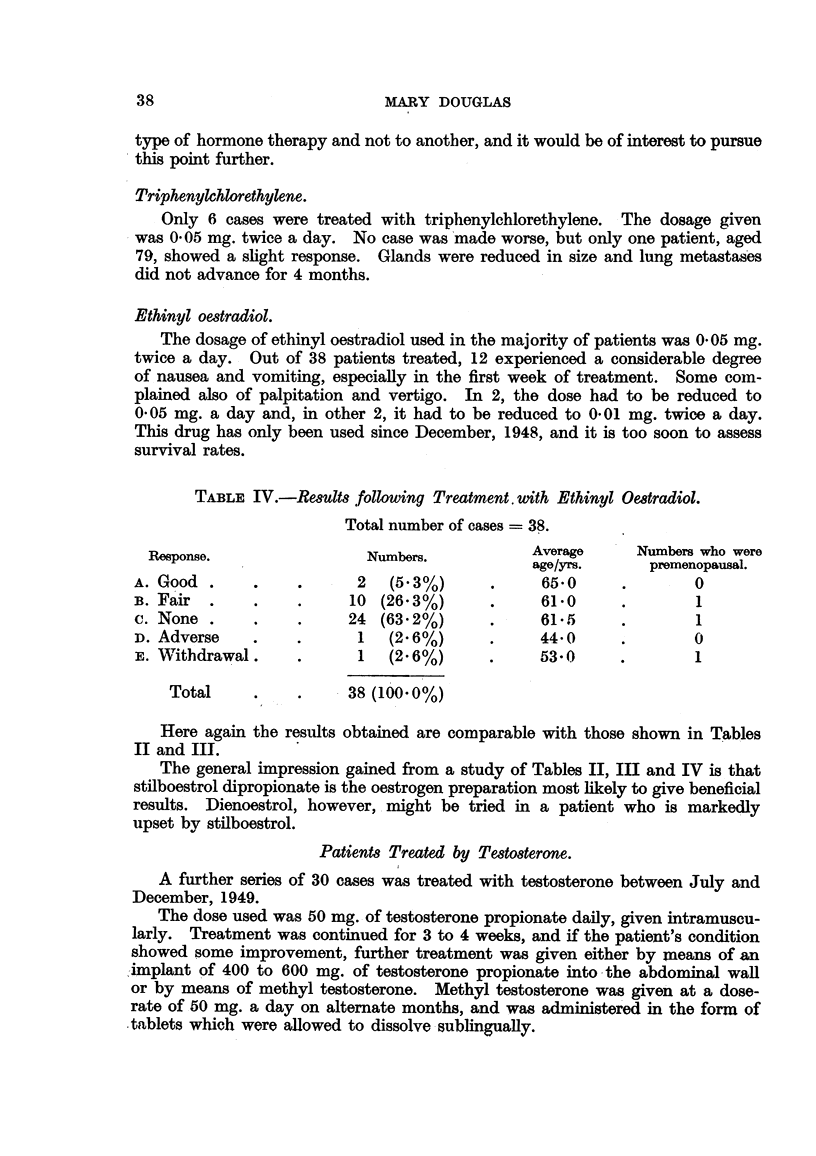

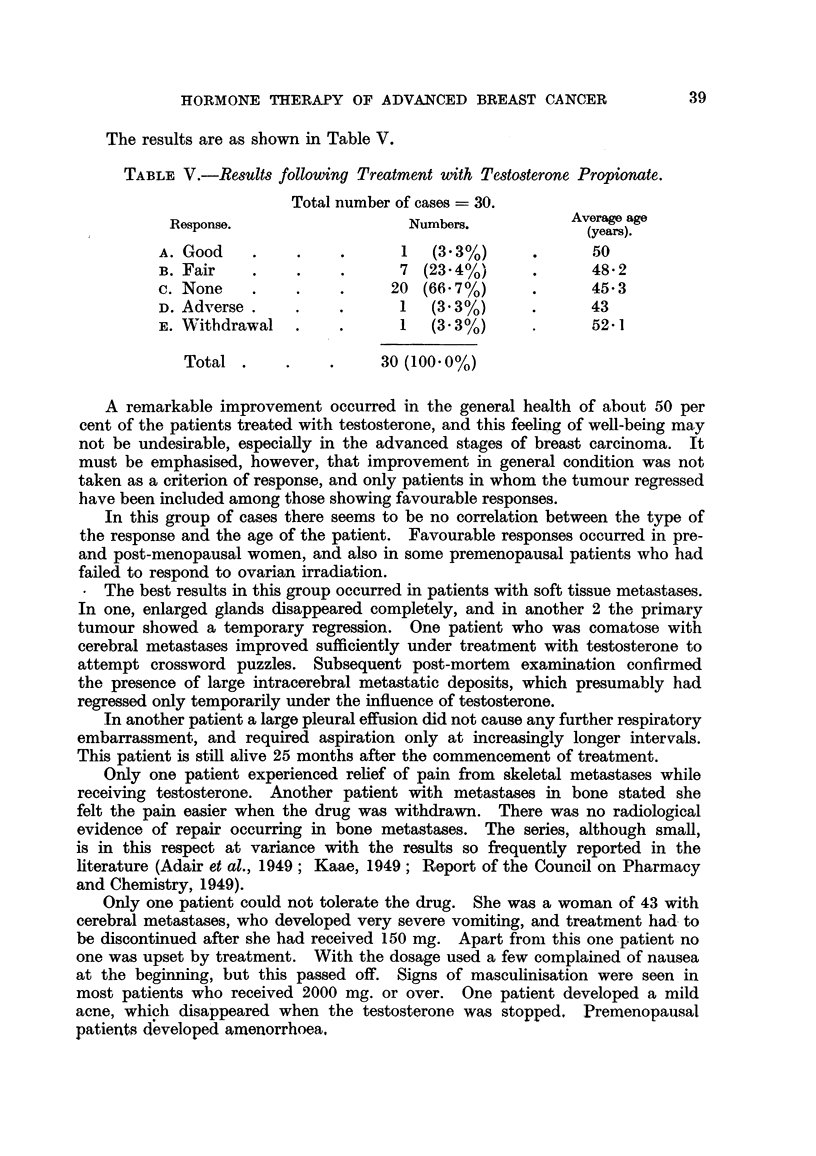

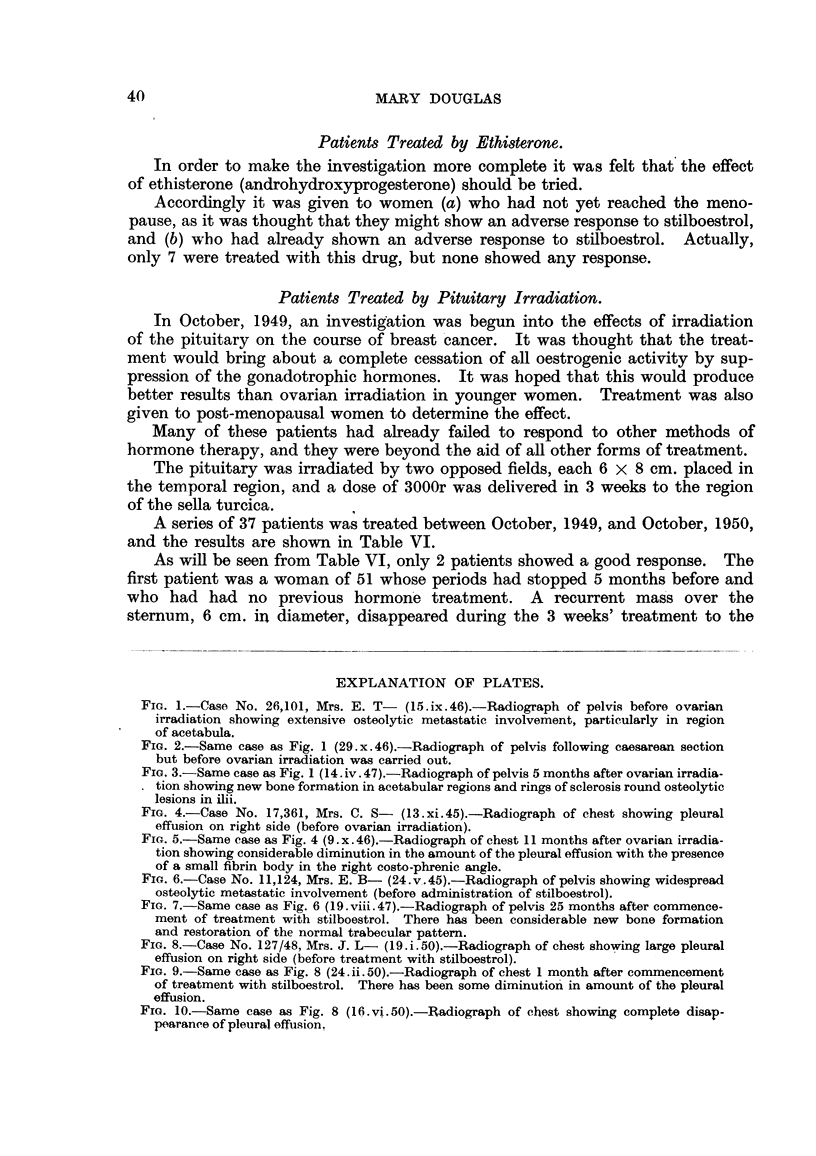

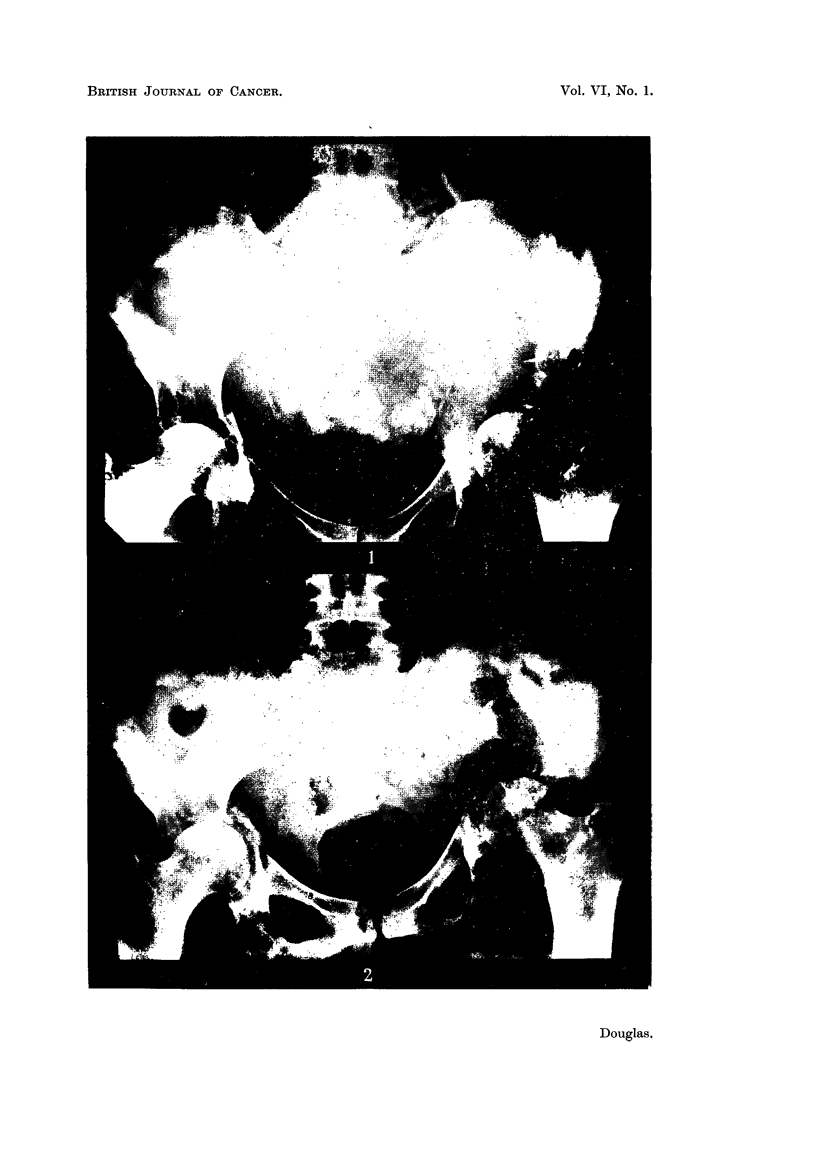

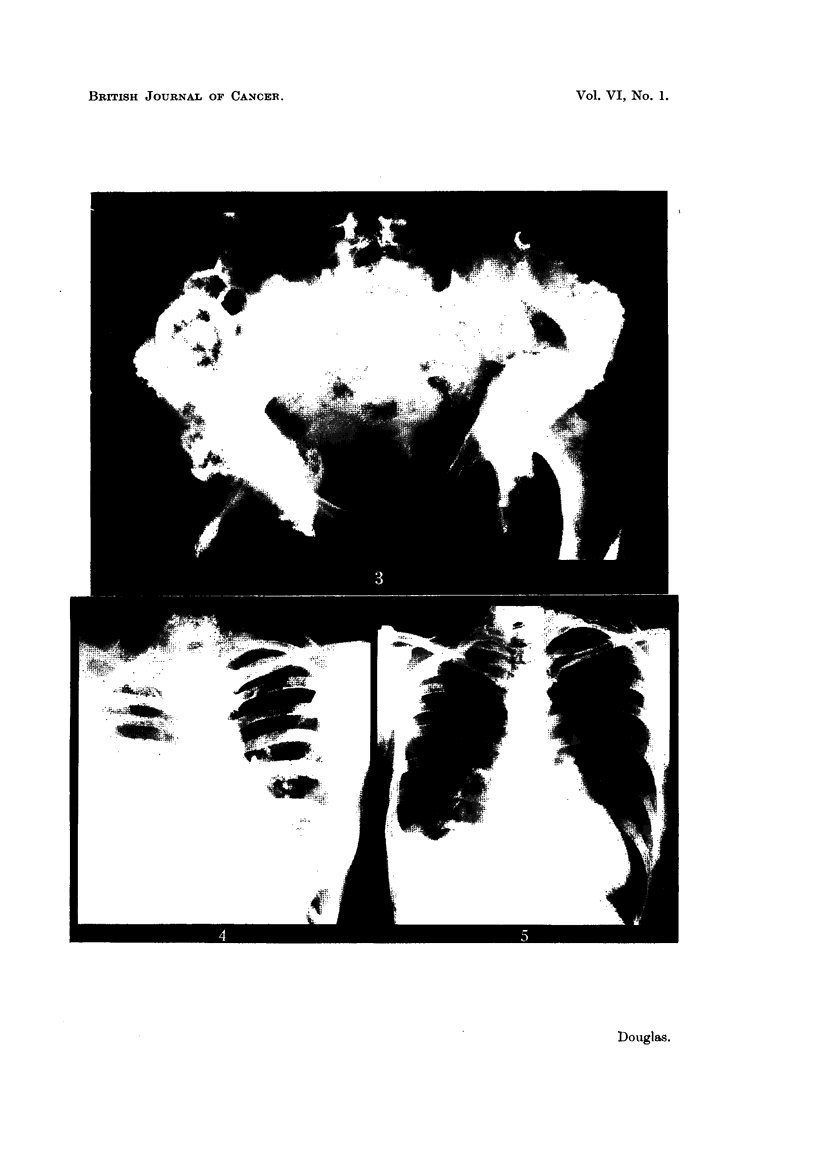

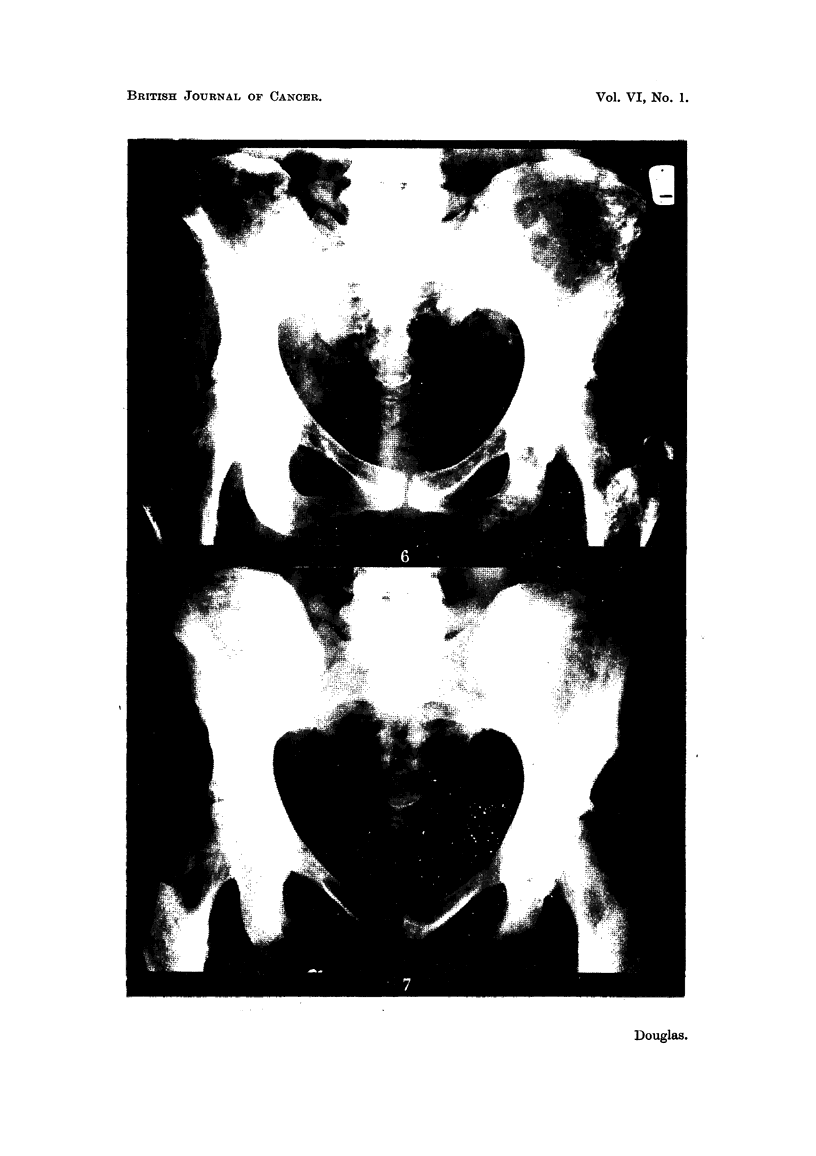

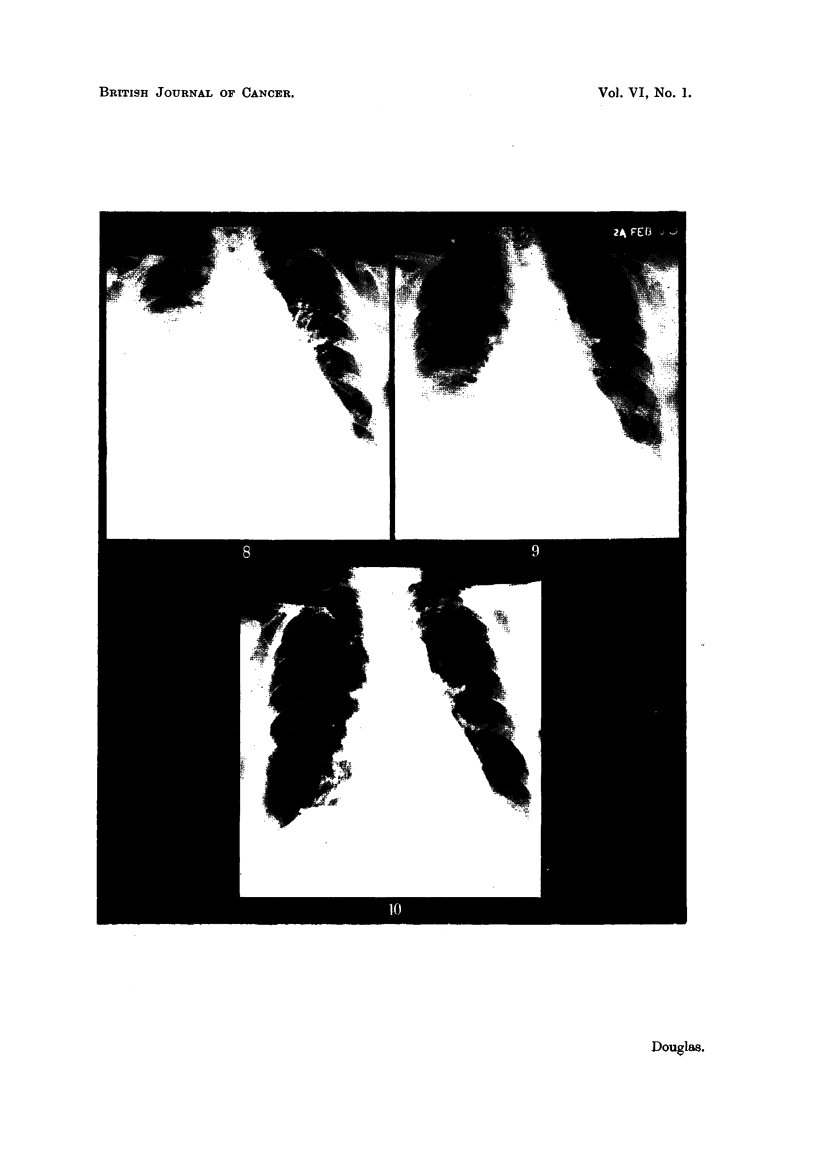

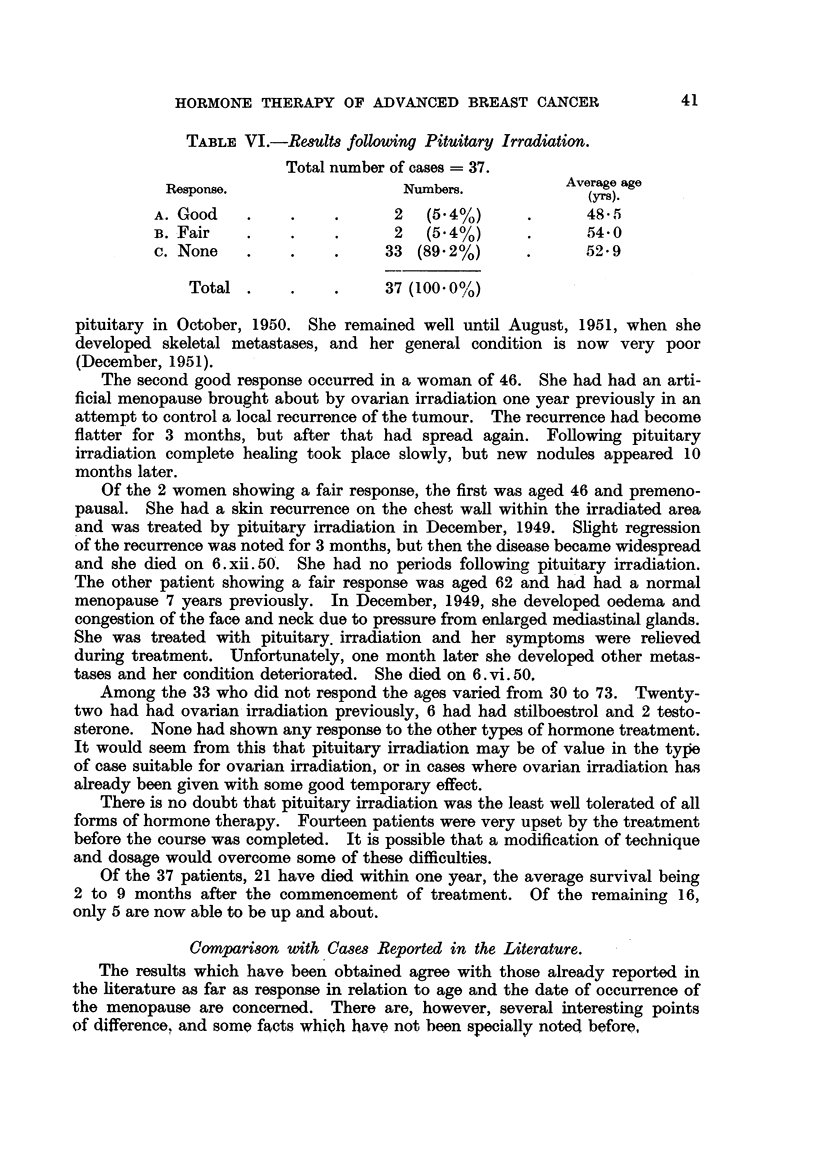

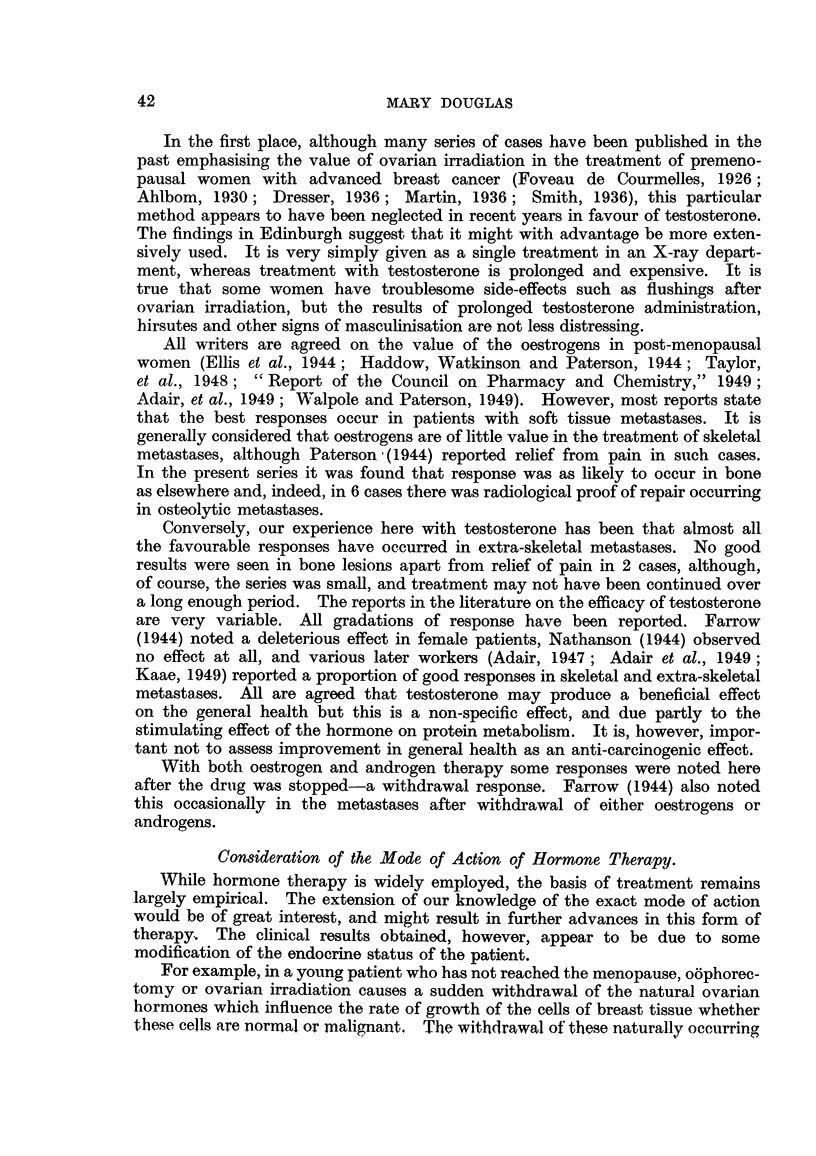

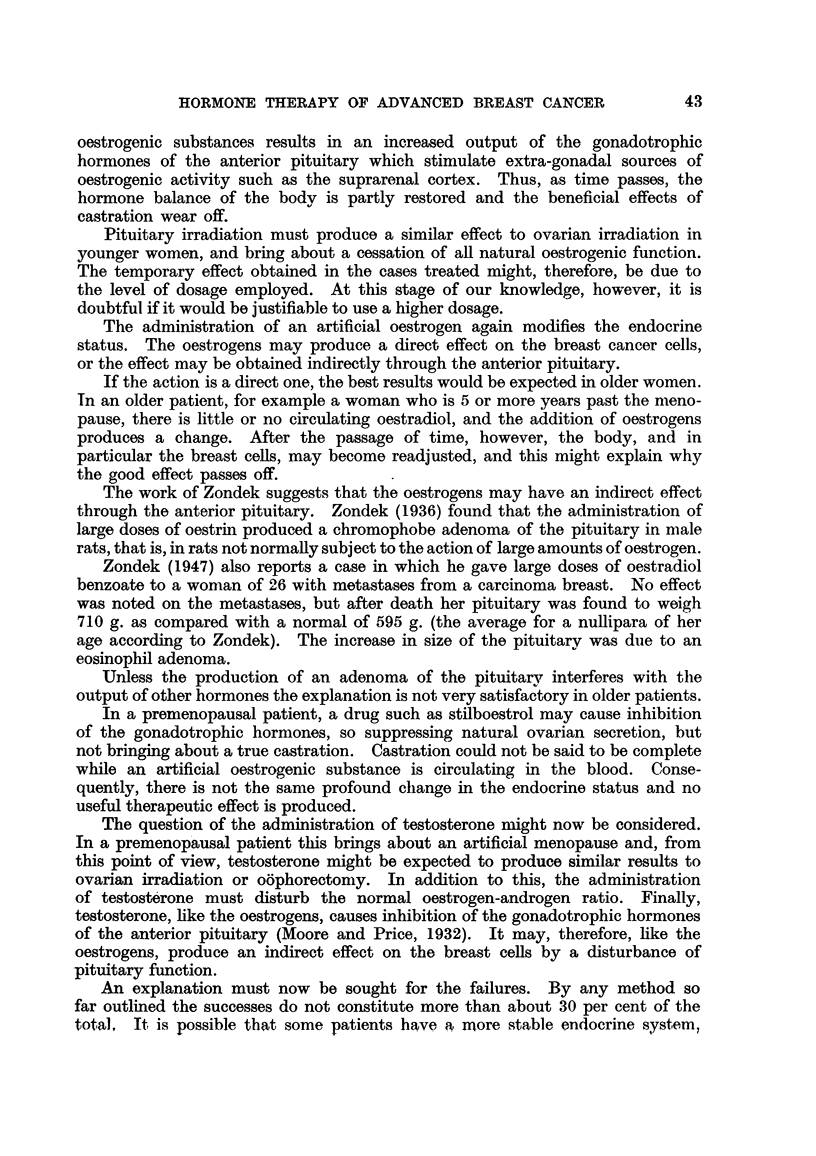

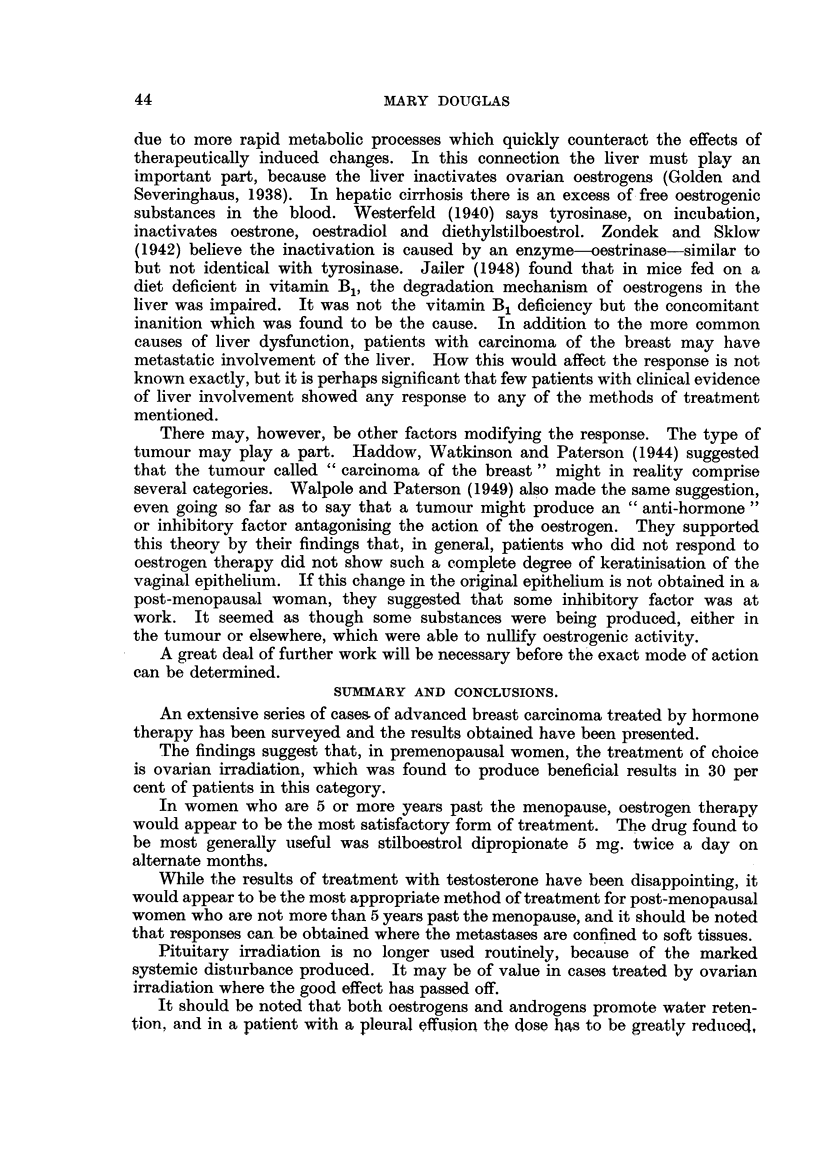

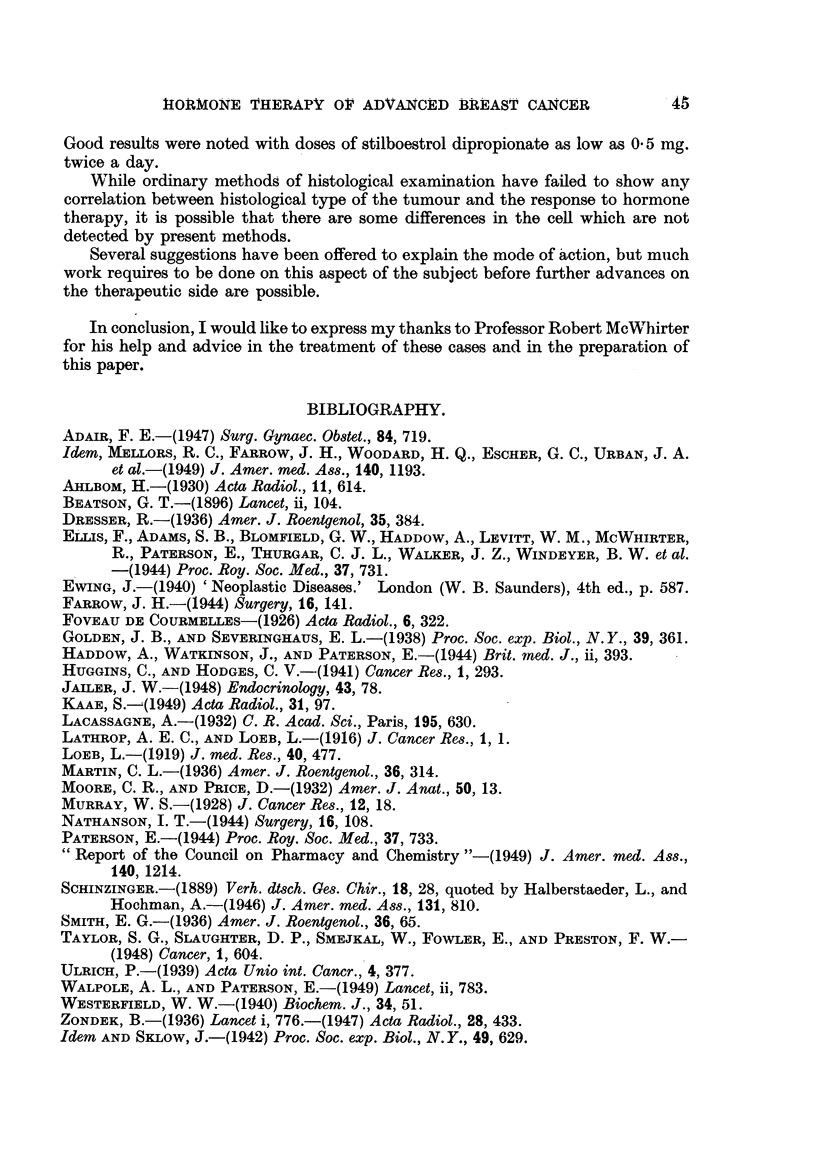


## References

[OCR_00954] Edkins N., Murray M. M. (1928). The effect of alcohol on the absorption of glucose from the alimentary tract: Part II.. J Physiol.

[OCR_00974] WALPOLE A. L., PATERSON E. (1949). Synthetic oestrogens in mammary cancer.. Lancet.

